# Characterization of AmpC, CTX-M and MBLs types of β-lactamases in clinical isolates of *Klebsiella pneumoniae* and *Escherichia coli* producing Extended Spectrum β-lactamases in Kerman, Iran

**DOI:** 10.5812/jjm.8756

**Published:** 2014-02-01

**Authors:** Shahla Mansouri, Davood Kalantar Neyestanaki, Mostafa Shokoohi, Shahnaz Halimi, Reza Beigverdi, Fereshteh Rezagholezadeh, Ali Hashemi

**Affiliations:** 1 Department of Microbiology, School of Medicine, Kerman University of Medical Sciences, Kerman, IR Iran; 2 Department of Microbiology, School of Medicine, Tehran University of Medical Sciences. Tehran, IR Iran; 3 Physiology Research Center, Kerman University of Medical Sciences, Kerman, IR Iran; 4 Department of Microbiology, School of Medicine, Shahid Beheshti University of Medical Sciences. Tehran, IR Iran

**Keywords:** Antibiotic Resistance, ESBLs, AmpC-β-lactamases, *bla*_*CTX-M*_, *Escherichia coli*, *Klebsiella pneumoniae*

## Abstract

**Background:**

Extended spectrum β-lactamases (ESBLs) and AmpC β-lactamases enzyme are major sources of resistance to β-lactam antibiotics especially in *Enterobacteriaceae* such as *Escherichia coli *and *Klebsiella pneumoniae*. Increasing frequency of the co-existence of ESBLs with AmpC-β-lactamases in bacteria is a serious threat for treating bacterial infections.

**Objectives:**

The aim of this study was to determine the presence of AmpC and CTX-M types of β-lactamases in clinical isolates of *E. coli* and *K. pneumoniae* producing ESBLs.

**Materials and Methods:**

Resistance to different antibiotics was determined using the standard disk diffusion method. ESBLs, MBLs and AmpC-β-lactamases were detected by the combination double disk test (CDDT) method and polymerase chain reaction (PCR) was used to determine *bla*_*CTX-M*_ genes in the ESBLs and AmpC positive isolates.

**Results:**

The prevalence of ESBLs and AmpC-β-lactamase producer isolates was 181 (43.8%) and 133 (37.2%), respectively. The prevalence of *bla*_*CTX-M*_ among isolates was 61 (14.7%).

**Conclusions:**

Outbreak of isolates co-expressing AmpC-β-lactamases and ESBLs can cause serious problems in the future, regarding the treatment of infections caused by these common enteric pathogens.

## 1. Background

*Escherichia coli* and *Klebsiella pneumoniae* are important causes of different bacterial infections, including cholecystitis, bacteremia, cholangitis, urinary tract infections (UTI), neonatal meningitis and pneumonia (1, 2). The *β-lactams* antibiotics are one of the treatment choices for these bacterial infections (3). One of the main mechanisms of resistance to *β-lactams* antibiotics is via the actions of β-lactamase enzymes (1). Extended spectrum β-lactamases (ESBLs) are *β*-lactamases that hydrolyze extended-spectrum cephalosporins such as cefotaxime and ceftazidime(4). Metallo-β-lactamases (MBLs) are a diverse group of β-lactamases that are active not only against the cephalosporins but also against the carbapenems (5). According to Ambler classification, AmpC-β-lactamases are an important group of class C β-lactamases that hydrolize penicillins, extended-spectrum cephalosporins, cephamycins and aztreonam, however they can't be inhibited by β-lactamase inhibitors such as clavulanate, sulbactam and tazobactam, but are inhibited by phenylboronic acid and cloxacillin (6, 7).

ESBLs and AmpC β-lactamases may co-exist, thus their detection is difficult because they mask each other and cause an increase in the minimum inhibitory concentrations for β-lactamas antibiotics (7). CTX-M type of the β-lactamase enzymes is a ESBLs type that is widely reported in *Enterobacteriaceae* such as *E. coli and K. pneumoniae*. This enzyme is the predominant ESBLs type in some countries (8). The CTX-M enzymes are usually encoded by genes that are carried on the plasmid and have greater activity against cefotaxime than other oxyimino-cephalosporins such as ceftazidime (9).

## 2. Objectives

The aim of this study was to determine the presence and prevalence of MBLs, AmpC and CTX-M types of β-lactamases among clinical isolates of *E. coli* and *K. pneumoniae* producing ESBLs in Kerman, Iran.

## 3. Materials and Methods

### 3.1. Bacterial Strains

In total, 413 consecutive non-duplicate *E. coli *(n = 338) and* K. pneumoniae *(n = 75) were isolated from clinical specimens including blood, urine and body fluids of patients admitted to three major hospitals (Afzali Poor, Kashani and Bahonar) located in three different regions of Kerman, southeast Iran from November 2007 to July 2008. The isolates were identified by their cultural characteristics and reactions to standard biochemical tests.

### 3.2. Antimicrobial Susceptibility Testing

The antibiotic resistance pattern of isolates was determined by using the disk diffusion method according to the Clinical and Laboratory Standard Institute (CLSI) guidelines (10). The antibiotics tested were ceftizoxime (30 μg), cefotaxime (30 μg), ceftazidime (30 μg), cephalexin (30 μg), amoxicillin (30 μg), imipenem (10 μg), cefepime (30 μg), cefoxitin (30 μg), gentamycin (30 μg), tetracycline (30 μg), trimethoprim/sulfamethoxazole (30 μg), nalidixic acid and ciprofloxacin (30 μg) (Himedia, India). *E. coli* ATCC 25922 and *K. pneumoniae* 700603 were used as quality control strains for antimicrobial susceptibility testing.

### 3.3. Detection of ESBLs Producing Isolates

ESBLs producing isolates were detected using the combination double disk test (CDDT) as a standard disk diffusion assay on Mueller Hinton agar (Himedia, India) (11). ESBLs presence was assayed using the following antibiotic disks: ceftazidime (CAZ) (30 μg), ceftazidime (30 μg) plus clavulanic acid (CA) (10 μg), cefotaxime (CTX) (30 μg), cefotaxime (30 μg) plus clavulanic acid (10 μg), and cefpodoxime (30 μg), cefpodoxime (30 μg) plus clavulanic acid (10μ g) (MAST Chemical Co, England). The disk with CA and without CA was placed on the inoculated surface of the Mueller–Hinton agar (Himedia, India) plate by the standard disk diffusion method. The plates were then incubated overnight at 37°C in ambient air. An increase of ≥ 5 mm in zone diameter of CAZ, CPD and/or CTX tested in combination with CA (CAZ-CA, CPD-CA and/or CTX-CA) versus CAZ, CPD and/or CTX alone was considered positive for ESBLs. *E. coli* ATCC 25922 and *K. pneumoniae* 700603 were used as control strains for detection of ESBLs producing isolates.

### 3.4. Determination of AmpC Producing Isolates

AmpC phenotype was specified by means of compound disk using cefoxitin (FOX), cefepime (CPM), CTX and CAZ disks (30 µg) alone and in combination with 400 µg of phenylboronic acid (BA) (SIGMA-ALDRICH, Fluka, China). The disk with BA and without BA was placed on the inoculated surface of the Mueller–Hinton agar plate (Himedia, India) by the standard disk diffusion method. The plates were then incubated overnight at 37°C in ambient air. An increase of ≥ 5 mm in zone diameter of CAZ, CTX, FOX and/or CPM tested in combination with BA (CAZ, CTX, FOX–BA and/or CPM –BA) versus CAZ, CTX, FOX and/or CPM alone was considered positive for AmpC β-lactamases (12).

### 3.5. Determination of MBLs Producing Isolates

Disks containing imipenem and imipenem with 5 µL EDTA (0.5 M) (SIGMA-ALDRICH, China) were used for determination of the presence of MBLs. An increase of ≥ 7 mm in zone diameter of imipenem tested in combination with EDTA versus imipenem alone was considered as MBLs positive (13).

### 3.6. DNA Extraction and Amplification 

Total DNA template was extracted as described previously (13). The primers used for PCR amplification were *bla*_CTX-M_ F-5^'^- CGC TTT GCG ATG TGA AG-3^'^ and *bla*_CTX-M_ R-5^'^- ACC GCG ATA TCG TTG GT-3^'^ (14). The PCR reactions were carried out in a Primus thermo cycler by using the PCR Master Kit (Cinna Clone Inc., Iran) according to the manufacturer guideline. PCR condition was as follows: initial denaturation at 95°C for 4 minutes followed by 30 cycles of denaturation at 95°C for 1 minute, annealing for 1 minute at 60°C, extension at 72°C for 1 minute. The final extension step was continued for another 5 minutes at 72°C.

### 3.7. Statistical Analysis

Statistical analysis was carried out using the SPSS 15 statistical software. We used the Chi-squared analysis for comparison of data. 

## 4. Results

A total of 338 strains of *E. coli* (urine= 316, blood = 14, other body fluids = 8) and 75 strains of *K. pneumoniae* (urine = 57, blood = 13, other body fluids = 5) were collected from three hospitals in Kerman, Iran. The rate of resistance to different antibiotics in* K. pneumoniae* and *E. coli* are shown in Table 1. 

**Table 1.** Rate of resistance to different antibiotics

Antibiotics	*E. coli *No. (%)	*K. pneumoniae*, No. (%)
**IMP^a^**	0 (0)	2 (2.6)
**CT^a^**	43 (12.7)	18 (24)
**CTX^a^**	105 (31)	25 (33)
**GM^a^**	133 (39.3)	48 (64)
**CPM** ^ a^	139 (41.1)	27 (36)
**CP^a^**	152 (44.9)	21 (28)
**FOX^a^**	156 (46.1)	29 (38)
**CAZ** ^ a^	188 (55.6)	52 (69)
**CN^a^**	213 (63)	54 (72)
**NA^a^**	243 (71.9)	30 (40)
**TET^a^**	283 (83.7)	40 (53)
**AMX^a^**	309 (91.4)	63 (84)
**SXT ** ^a^	316 (93.4)	35 (47)

^a^ Abbreviations: AMX, amoxicillin; CN, cefalexin; CT, ceftizoxime; CAZ, ceftazidime; CPM, cefepime; CTX, cefotaxime; CP, ciprofloxacin; FOX, cefoxitin; GM, gentamycin; IMP, imipenem; STX, trimethoprim/sulfamethoxazole; TET, tetracycline; NA, nalidixic acid

In this study, only two* K. pneumoniae* were resistant to imipenem, which were isolated from blood. Eighty-four percent of the isolates of *K. pneumoniae* were resistant to at least one antibiotic and 12 (16%) isolates were susceptible to all tested antibiotics.

**Figure 1.**
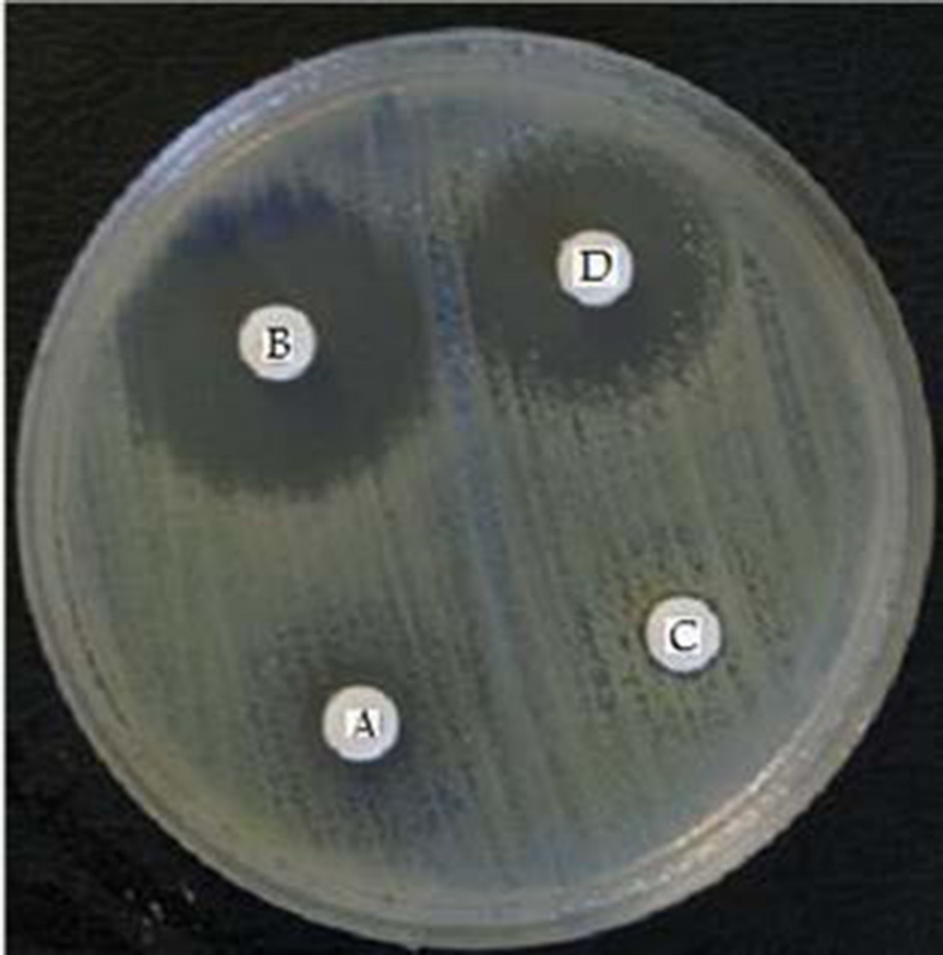
Extended spectrum β-Lactamases producing *K*.* pneumoniae* (A = Cefotaxime B = Cefotaxime + Clavulanic acid) C = Ceftazidime D = Ceftazidime + Clavulanic acid) Clavulanic acid inhibited an extended spectrum of β-lactamases with the occurrence of a growth inhibition zone.

**Figure 2.**
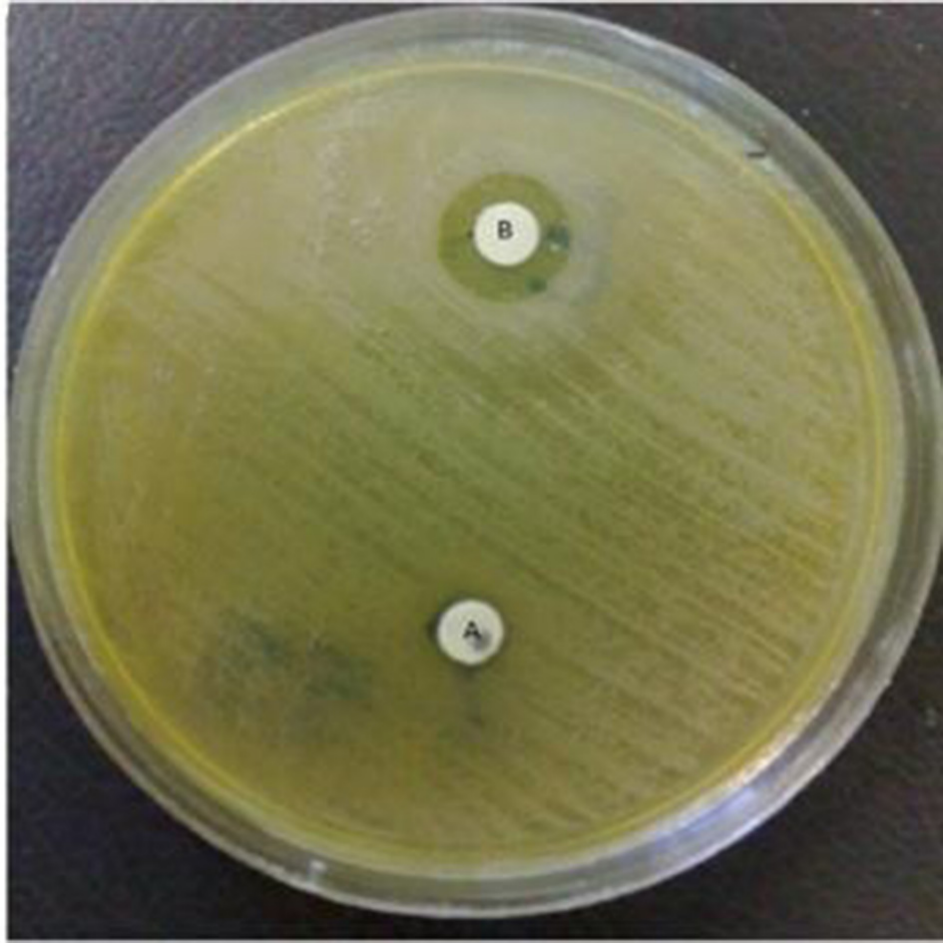
MBLs Producing *K*.* pneumoniae* A = Imipenem, B = Imipenem +EDTA (.0.5 M).

More than 50 % of *K. pneumoniae* isolates were resistant to amoxicillin, cephalexin, ceftazidime and gentamycin. Among 75 *K. pneumoniae* isolates 33 (44%) produced ESBLs, 21 (28%) produced AmpC β-lactamases and 1 (1.3%) produced MBLs (Figures 1 and 2). 

Simultaneous production of ESBLs and AmpC β-lactamases were observed in 21 (28%) isolates of *K. pneumoniae* (Table 2). More than 321 (95%) of the isolates of *E. coli* were resistant to at least one of the following antibiotics, amoxicillin, cephalexin, tetracycline, trimethoprim/sulfamethoxazole, nalidixic acid, ciprofloxacin and gentamicin. Out of the 338 *E. coli* isolates, 148 (43.7%) produced ESBLs and 133 (39.3%) produced AmpC β-lactamases. Six isolates of *E. coli* only produced AmpC β-lactamases. Co-existence of ESBLs and AmpC β-lactamases were observed in 133(39.3%) isolates (Table 3). 

**Table 2.** Antibiotic resistance patterns of *K. pneumoniae* isolates producing ESBLs and AmpC

Antibiotic Resistance Patterns ^a^	Isolates No.				
	Blood	Urine	AmpC	*blaCTX-M*	Others
**CP, CTX, CAZ, CT, AMX, GM, NA, TET, CN, SXT, FOX, CPM**	-	3	3	2	-
**CTX, CAZ, CT, AMX, GM, NA, TET, CN, SXT, FOX, CPM**	1	1	1	1	-
**CP, CAZ, CT, AMX, GM, NA, TET, CN, SXT, FOX, CPM**	-	2	2	-	-
**CP, CTX, CAZ, CT, AMX, GM, NA, TET, CN, SXT, FOX**	-	2	2	-	-
**CTX, CAZ, AMX, GM, NA, TET, CN, SXT, FOX, CPM**	-	1	1	1	-
**CP, CTX, CAZ, AMX, GM, TET, CN, SXT, FOX, CPM**	-	1	-	-	-
**CP, CAZ, CT, AMX, GM, NA, TET, CN, SXT, FOX**	-	1	-	-	-
**CP, CTX, CAZ, AMX, TET, CN, SXT, FOX, CPM**	-	1	1	-	-
**CTX, CAZ, AMX, GM, NA, CN, SXT, FOX, CPM**	-	-	-	-	1
**CTX, CAZ, AMX, GM, CN, SXT, FOX, CPM**	6	2	9	8	1
**CTX, CAZ, AMX, NA, CN, SXT, FOX, CPM**	-	1	1	1	-
**IMP, CTX, CT, AMX, GM, CN, FOX, CPM**	1	-	-	1	-
**CAZ, AMX, GM, TET, CN, SXT, FOX, CPM**	1	-	-	-	-
**CTX, CAZ, AMX, GM, CN, FOX, CPM**	-	1	-	1	-
**CAZ, AMX, GM, CN, SXT, FOX, CPM**	-	1	1	-	-
**CTX, AMX, GM, CN, SXT, FOX, CPM**	1	-	-	-	-
**CTX, CAZ, AMX, CN, FOX, CPM**	-	1	-	-	-
**CTX, CAZ, CN, SXT, FOX, CPM**	-	1	-	-	-
**IMP, CT, AMX, GM, TET, CN**	1	-	-	-	-
**CTX, CAZ, AMX, CN**	-	1	-	1	-

^a^ For abbreviations please refer to Table 1.

All ESBLs and AmpC producer isolates were multi drug resistant and showed resistance to four different antibiotics (Tables 2 and 3).

**Figure 3.**
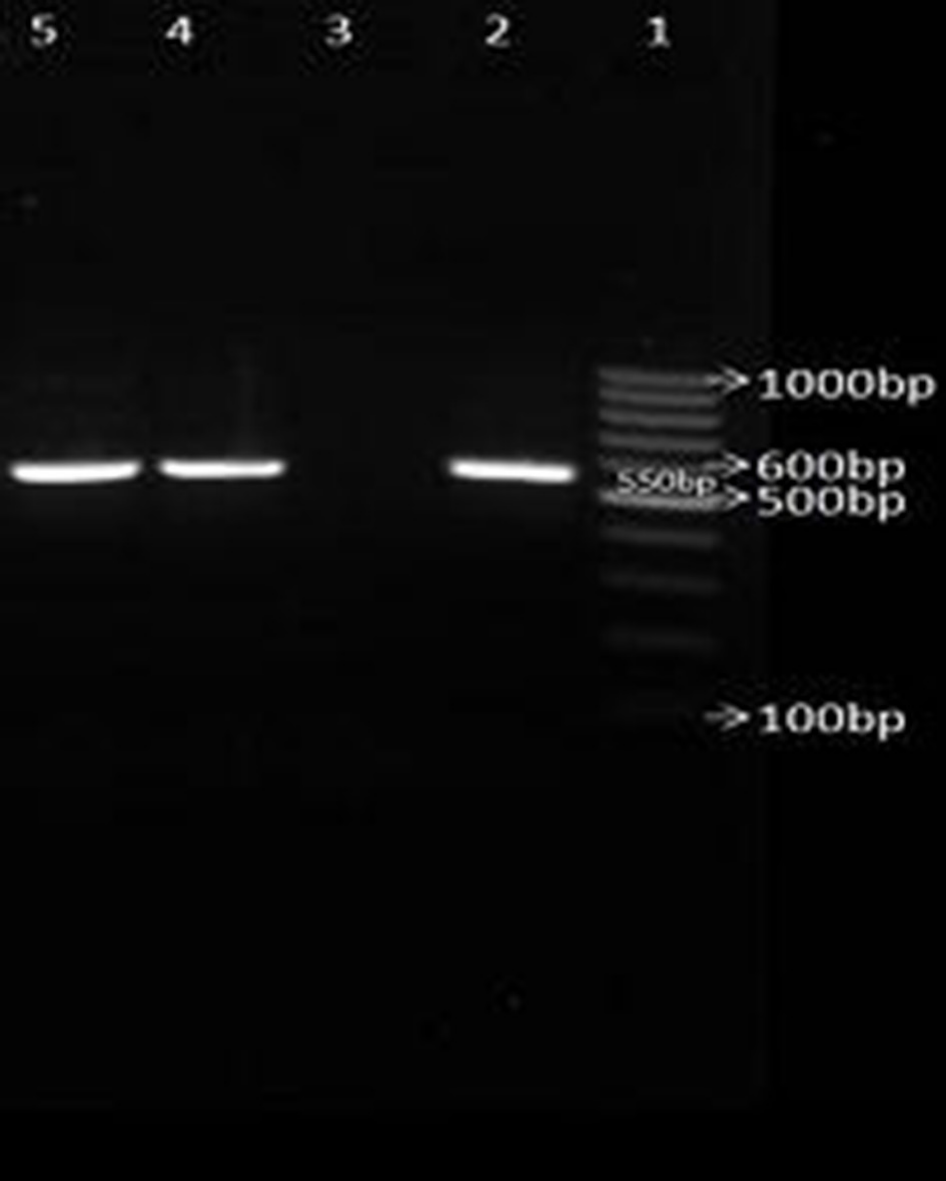
Electrophoresis of PCR products for amplifying *blaCTX-M* broad spectrum β-lactamases genes. No.1: DNA marker (100 bp), No 2: positive control for *blaCTX-M*, No. 3: negative control No. 4,5: isolates with *blaCTX-M* (550 bp) gene.

None of the AmpC β-lactamases producing isolates were resistant to imipenem. From a total of 133 AmpC positive isolates, 19 were detected with cefoxitin, 81 were detected with cefepime, 82 were detected with cefotaxime and 91 isolates were detected with ceftazidime. The prevalence rate of *bla*_CTX-M_ gene among ESBLs and AmpC producing isolates of *K. pneumoniae* and *E. coli* were 16 (21.3%) and 45 (13.3%) respectively (Figure 3). 

**Table 3.** Antibiotic resistance patterns of *E.* *coli* isolates producing ESBLs

	Isolates No.				
Antibiotic Resistance Patterns ^a^	Blood	Urines	AmpC	*blaCTX-M*	Other
**CP, CAZ, CT, AMX,GM, NA,TET, CN, SXT, FOX, CPM, CTX**	1	10	11	6	-
**CAZ, CT, AMX, GM, NA, TET, CN, SXT, FOX, CPM, CTX**	-	2	-	1	-
**CP, CAZ, CT, AMX, NA,TET, CN, SXT, FOX, CPM, CTX**	1	4	4	2	-
**CP, CAZ, CT, AMX,GM, NA, CN, SXT, FOX, CPM, CTX**	-	-	1	-	1
**CP, CAZ, AMX,GM, NA,TET, CN, SXT, FOX, CPM, CTX **	4	16	16	6	-
**CP, CAZ, CT, AMX, GM, NA,TET, CN, SXT, FOX, CPM**	-	1	2	-	1
**CAZ, CT, AMX, GM, TET, CN, SXT, FOX, CPM, CTX**	-	1	1	1	-
**CAZ, AMX, GM, NA, TET, CN, SXT, FOX, CPM, CTX**	-	2	3	2	1
**CAZ, CT, AMX, NA, TET, CN, SXT, FOX, CPM, CTX**	-	2	1	1	-
**CP, CAZ, AMX, NA,TET, CN, SXT, FOX, CPM, CTX**	-	14	13	4	-
**CP, AMX,GM, NA,TET, CN, SXT, FOX, CPM, CTX**	-	1	2	2	1
**CAZ, CT, AMX, GM, NA, TET, CN, SXT, FOX,CTX**	-	1	1	-	-
**CP, CAZ, AMX,GM, NA,TET, CN, SXT, FOX, CPM**	-	4	4	-	-
**CAZ, AMX, GM, TET, CN, SXT, FOX, CPM, CTX**	-	1	1	-	-
**CAZ, AMX, NA, TET, CN, SXT, FOX, CPM, CTX**	1	7	7	3	-
**CAZ, CT, AMX, TET, CN, SXT, FOX, CPM, CTX**	-	1	1	-	-
**CP, CAZ, AMX , NA, CN, SXT, FOX, CPM, CTX**	-	2	1	1	-
**CP, CAZ, AMX, TET, CN, SXT, FOX, CPM, CTX**	-	1	1	-	-
**CAZ, AMX, GM, NA, TET, CN, SXT, FOX, CPM**	-	3	3	-	-
**CAZ, CT, AMX, NA, TET, CN, SXT, FOX, CPM**	-	1	-	-	-
**CAZ, CT, AMX, NA, CN, SXT, FOX, CPM, CTX**	-	4	4	3	-
**CAZ, CT, AMX, NA, TET, CN, SXT, FOX, CPM**	-	1	-	-	-
**CP, CAZ, AMX, NA,TET, CN, SXT, FOX, CTX**	-	1	-	-	-
**CP, CAZ, AMX, NA,TET, CN, SXT, FOX, CPM**	-	3	3	-	-
**CAZ, AMX, NA, TET, SXT, FOX, CPM, CTX**	-	3	3	2	-
**CP, CAZ, AMX,GM, NA,TET, CN, SXT, FOX**	-	1	1	-	-
**CAZ, AMX, TET, CN, SXT, FOX, CPM, CTX**	-	3	3	-	-
**CP, CAZ,GM, NA,TET, CN, SXT, FOX, CPM**	-	1	1	-	-
**CAZ, AMX, GM, CN, SXT, FOX, CPM, CTX**	-	3	2	1	-
**CAZ, AMX, GM, CN, SXT, FOX, CPM, CTX**	-	1	1	-	-
**CAZ, AMX, GM, TET, CN, FOX, CPM, CTX**	-	1	1	1	-
**CAZ, AMX, GM, TET, CN, SXT, FOX, CPM**	1	2	2	-	-
**CAZ, AMX, NA, TET, CN, SXT, FOX, CPM**	1	2	2	-	-
**CAZ, AMX, GM, NA, TET, CN, SXT, FOX**	-	2	-	-	-
**CP, AMX, NA,TET, SXT, FOX, CPM,CTX**	-	1	1	1	-
**CP, AMX, NA,TET, CN, FOX, CPM, CTX**	-	-	1	1	1
**CAZ, CT, AMX, NA, TET, CN, SXT, FOX**	-	2	2	-	-
**CAZ, CT, AMX, NA,CN, SXT, FOX,CTX**	-	1	1	-	-
**CP, CAZ, NA,TET, CN, SXT, FOX, CPM**	-	1	1	-	-
**CAZ, AMX, GM, SXT, FOX, CPM, CTX**	-	2	2	2	-
**CP, CAZ, AMX,GM, NA,TET, CN, SXT**	-	2	-	-	-
**CAZ, AMX, CN, SXT, FOX, CPM, CTX**	-	2	2	1	-
**CAZ, AMX, TET, CN, SXT, FOX, CPM**	-	3	3	-	-
**CAZ, AMX, GM, CN, SXT, FOX, CPM**	1	1	2	-	-
**CAZ, AMX, GM, CN, FOX, CPM, CTX**	1	-	1	-	-
**CAZ, AMX, GM, TET, CN, SXT, FOX**	-	1	-	-	-
**CAZ, AMX, NA, TET, CN, FOX, CPM**	-	1	1	-	-
**CAZ, AMX, NA, TET, CN, SXT, CTX**	-	1	-	-	-
**CAZ, AMX, NA, TET, CN, SXT, FOX**	-	1	1	-	-
**CP, CAZ, AMX, NA, CN, SXT, FOX**	-	1	1	-	-
**CAZ, AMX, TET, FOX, CPM, CTX**	-	2	2	2	-
**CAZ, AMX, CN, SXT, FOX, CPM**	-	1	1	-	1
**CAZ, AMX, TET, CN, SXT, CTX**	-	1	-	-	-
**CAZ, AMX, GM, CN, FOX, CPM**	1	-	1	-	-
**CAZ, AMX,TET, CN, FOX, CPM**	-	1	1	-	-
**CAZ, AMX, GM, CN, SXT, CTX**	-	1	-	1	-
**CAZ, AMX, NA, TET, CN, CTX**	-	1	-	1	-
**CAZ, AMX, NA, TET, CN, SXT**	-	1	-	-	-

^a^ For abbreviations please refer to Table 1.

## 5. Discussion

Appearance of β-lactamase enzymes among Gram-negative bacteria especially those that have a key role in hospital infections, is an important concern. The prevalence of strains possessing several resistance enzymes concomitantly has caused serious problems for the treatment and the diagnosis of such strains (7). Compared to the results of previous studies in 2002, the percentages of multi-resistance isolates increased in *E. coli* (15). In general, ESBLs and AmpC β-lactamases producing isolates are susceptible to imipenem (4). In this study, similar to other reports, more than 99% of isolates were susceptible to imipenem. However, two isolates in our study were resistant to imipenem and one of them was a MBLs producer isolate. Fortunately, carbapenem resistance is still very rare among *K. pneumoniae* and *E. coli* in Iran (16-18). 

In the current study, the 28 (*K. pneumoniae* =6 and* E. coli* =22) isolates of *K. pneumoniae* and *E. coli*, which were resistant to cefoxitin, ceftazidime, cefotaxim and cefepime, were not inhibited by clavulanic acid and phenylboronic acid. These results can partially be explained by the concomitant presence of several resistance mechanisms in these isolates. It may also be inferred that the procedures available for the detection of β-lactam resistance phenotypes are no longer practical. Getzlaff et al. reported that cefoxitin and cefoxitin plus phenylboronic acid had 95% specificity for phenotypic AmpC detection but in this study only 14.2% (19/133) of AmpC producing isolates were detected by this method, which may be related to the presence of other mechanisms of resistance to cefoxitin in this area (19). 

Furthermore, in our study the AmpC β-lactamases were also the source of one of the resistance mechanisms to ceftazidime, cefotaxim and cefepime. In this study, the prevalence of the phenotypes ESBLs and AmpC in *E. coli* was 43.7% and 39.3% and in* K. pneumoniae,* this was 44% and 28%, respectively. The prevalence of ESBLs in *E. coli* and *K. pneumoniae* as reported in this study is in agreement with that of an investigation from 2007 and 2009 in Tehran (16-18). This can partially show an invariant and uniform prevalence for ESBLs across Iran

According to the literature review, the prevalence of the phenotypes of AmpC has not yet been reported for the clinical isolates of *E. coli and K. pneumoniae* in Iran by the use of a phenylboronic acid disk. On the other hand, the prevalence rate of AmpC has been reported for South Korea (53%), India (47.3%) and Singapore (57%), which are higher than the prevalence rate in Iran (20, 21). There was a significant correlation between presence of *bla*_CTX-M_ and resistance to cefotaxime and ceftizoxime in our isolates (P < 0.05). CTX-M producing isolates amongst *E. coli and K. pneumoniae* have been reported from other parts of Iran such as Tehran and Kurdistan (16, 22, 23). Also, CTX-M producing isolates have also been identified in Korea, Italy, France, New Zealand, Egypt and other countries and carriers of CTX-M type of β-lactamases were subsequently hospitalized (21, 24-27). This enzyme has now become the most prevalent β-lactamases in hospitals and in the community (16).

The emergence of the co-production of ESBLs, AmpC and MBLs is increasing in Iran. The prevalence of isolates possessing several resistance enzymes concomitantly has caused serious problems for treatment and diagnosis. Consequently, regular epidemiological assessments on the drug resistance patterns of the isolates responsible for nosocomial infections and determination of the molecular resistance mechanisms can be useful for the empirical treatment of the infections and prevention of the drug resistance distribution.
